# Gibberellin derivative GA-13315 overcomes multidrug resistance in breast cancer by up-regulating BMP6 expression

**DOI:** 10.3389/fphar.2022.1059365

**Published:** 2022-11-30

**Authors:** Xianqiang Luo, Guohui Wang, Yuting Wang, Meichen Wang, Zhuomin Tan, Min Luo, Limei Zhang, Yan Song, Yinnong Jia, Hongyu Zhou, Chen Qing

**Affiliations:** ^1^ School of Pharmaceutical Science and Yunnan Key Laboratory of Pharmacology for Natural Products, Kunming Medical University, Kunming, China; ^2^ The Second People’s Hospital of Quzhou, Quzhou, China

**Keywords:** GA-13315 (GA5), multidrug resistance (MDR), doxorubicin (DOX), bone morphogenetic protein 6 (BMP6), ERK, P-gp

## Abstract

Chemoresistance represents a major obstacle in breast cancer treatment. Bone morphogenetic protein 6 (BMP6) was reported to participate in the occurrence and development of various tumors. In the present study, the results of transcriptome sequencing, qRT-PCR and western blot analysis revealed that BMP6 was down-regulated in multidrug resistant MCF-7/Adr breast cancer cells and BMP6 overexpression sensitized MCF-7/Adr cells to chemotherapeutic drugs, indicating that BMP6 downregulation was involved in the mechanisms of multidrug resistance (MDR) of MCF-7/Adr breast cancer cells. GA-13315 (GA5) is a new tetracyclic diterpenoid selected from a series of gibberellin derivatives. Here, we found that GA5 exhibited more potent anti-tumor activity in multidrug resistant MCF-7/Adr breast cancer cells and xenografts, indicating that GA5 could overcome MDR. Mechanistically, GA5 increased BMP6 expression, and BPM6 knockdown partially reversed the inhibitory effect of GA5 on cell proliferation. Furthermore, we found that ERK phosphorylation and P-gp expression were increased in MCF-7/Adr cells when compared with MCF-7 cells. Either overexpression of BMP6 or treatment the cells with GA5 significantly decreased ERK phosphorylation and P-gp expression, indicating that GA5 reversed MDR of MCF-7/Adr cells by upregulating BMP6, thereby inhibiting the activation of ERK signaling pathway and reducing P-gp expression. Collectively, our present study demonstrated that the MDR of MCF-7/Adr cells was closely related to the low expression of BMP6, and revealed the molecular mechanisms by which GA5 overcame MDR in breast cancer, providing evidence in supporting the development of GA5 to be a promising agent for overcoming MDR in clinical cancer therapy in the future.

## 1 Introduction

With the extensive clinical application of chemotherapeutic drugs, multidrug resistance (MDR) has become a major challenge in the successful treatment of cancers (Chen and Sikic 2012). During cancer chemotherapy, malignant cells become insensitive to drugs and develop drug resistance. MDR means that some tumor cells are not only resistant to chemotherapeutic drugs that they have been exposed to, but also resistant to other drugs that they have not been exposed to. The development of MDR greatly reduces the expected efficacy of clinical chemotherapy and is the main reason for the failure of chemotherapy in cancer treatments (Chen and Sikic 2012; Wu et al., 2014; Bukowski et al., 2020). Researchers have been investigating the underlying mechanisms involved in MDR and looking for drugs that can reverse or overcome tumor MDR (Dallavalle et al., 2020). But so far, no drug that can reverse or overcome tumor resistance has been successfully used in clinical practice. Therefore, it is still important to explore the molecular mechanism of tumor chemotherapy resistance, and to discover novel agents to reverse or overcome tumor resistance (Dallavalle et al., 2020).

**FIGURE 1:**
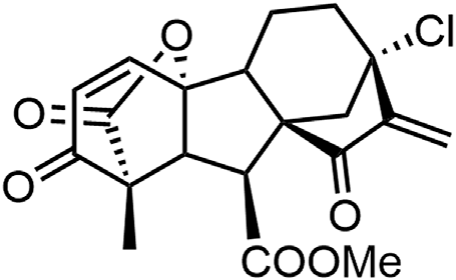
The structure of GA5.

GA-13315 (GA5), 13-Chlorine-3,15-dioxy-gibberellic acid methyl ester, is a gibberellin derivative with an α, β-unsaturated ketone moiety (Figure 1) (Yang et al., 2012; Shen and Tang 2017). Our previous study found that GA5 possessed excellent anti-tumor activity and low toxicity. Compared with the median lethal dose (LD_50_) on mice, the effective anti-tumor dose of GA5 was 170 times lower (Zhang et al., 2012; Shen and Tang 2017). In addition, GA5 exhibited characteristics of reversing MDR of human breast cancer cells (Mo et al., 2016). The study showed that the IC_50_ value of GA5 on MCF-7/Adr cells was nearly twice lower than that on MCF-7 cells, indicating that MCF-7/Adr cells were more sensitive to GA5 when compared with MCF-7 cells. Mechanistically, GA5 had an inhibitory effect on the ATPase activity of P-gp in drug-resistant cancer cells (Mo et al., 2016). Taken together, the above studies demonstrated that GA5 not only exhibited antitumor activity, but also had the potency to overcome breast cancer chemotherapy resistance, which is worthy of further exploration.

Bone morphogenetic protein 6 (BMP6) is a member of BMPs and belongs to the TGF-β superfamily. Studies showed that BMP6 was associated with the occurrence and development of various malignant tumors (Dai et al., 2005; Kimura et al., 2008; Wang et al., 2011; Lee et al., 2013). In breast cancer, as a tumor suppressor gene, BMP6 played an important role in the proliferation, differentiation and chemoresistance of breast cancer cells (Du et al., 2008; Du et al., 2009; Lian et al., 2013; Liu et al., 2014). Liu et al. reported that BMP6 expression was significantly downregulated in the majority of the primary breast cancer specimens when compared with that in the adjacent normal tissues. In the tumor tissues, the BMP6 mRNA or protein expression was significantly correlated with breast cancer tumor grade and ER and PR statuses. BMP6 expression in ER-negative breast cancer was obviously lower than that in ER-positive breast cancer (Lian et al., 2013). Furthermore, BMP6 was down-regulated in drug-resistant cells, and knockdown of BMP6 in MCF-7 cells enhanced the chemoresistance to doxorubicin, indicating that BMP6 was a critical regulator of breast cancer drug resistance. However, little is known about its mechanisms in breast cancer drug resistance.

In the present study, we found that GA5 had stronger inhibitory effect against multidrug-resistant MCF-7/Adr cells both *in vitro* and *in vivo*. In order to further investigate the mechanisms by which GA5 overcame MDR in MCF-7/Adr cells, transcriptome sequencing (RNA-Seq) was used to detect the transcriptome differences between parental cell line MCF-7 and multidrug resistant cell line MCF-7/Adr, and between GA5 treatment and non-GA5 treatment MCF-7/Adr cells. Base on the results of RNA-Seq, quantitative real-time polymerase chain reaction (qRT-PCR) and western blot, we found that BMP6 was down-regulated in multidrug resistant MCF-7/Adr cells when compared with the parental cell line MCF-7. Importantly, GA5 significantly up-regulated BMP6 mRNA and protein expressions. BMP6 knockdown partially reversed the anti-proliferation effect of GA5, suggesting that GA5 overcame MDR of MCF-7/Adr cells might be associated with upregulation of BMP6. Further mechanism studies showed that GA5 could inhibit the phosphorylation of ERK and decrease the expression of P-gp in both MCF-7/Adr cells and xenografts mouse model.

In summary, our present study provided evidence that BMP6 is a potential target for overcoming MDR in breast cancer, and GA5 could overcome MDR by increasing BMP6 expression, suggesting that GA5 had the potential in the treatment of multidrug resistant breast cancer.

## 2 Materials and methods

### 2.1 Chemicals and reagents

GA5 was provided by Prof. Jingbo Chen and Prof. Hongbin Zhang (Yunnan University, China). Doxorubicin (DOX) was purchased from Zhejiang Hanhui Pharmaceutical Co., Ltd. (China). Etoposide (VP-16) was purchased from Hainan Qilu Pharmaceutical Co., Ltd. (China). Epirubicin (EPI), Mitoxantrone (NVT), Hydroxycamptothecin (HCPT), protease inhibitor, phosphatase inhibitor, and MTT were purchased from Sigma-Aldrich (United States). TRIzol^®^ reagent, RevertAid First Strand cDNA Synthesis Kit and PowerUp^TM^ SYBR^TM^ Green Master Mix were purchased from Thermo Fisher Scientific Inc (United States). RPMI 1640 medium, fetal bovine serum (FBS), penicillin-streptomycin were purchased from Gibco (United States). All the other chemicals were purchased from commercial sources with analytical grade.

### 2.2 Cell lines and cell culture

The human breast cancer cell lines MCF-7 and MCF-7/Adr induced by DOX were purchased from the Cell Bank of Shanghai Institutes for Biological Sciences, Chinese Academy of Sciences. Both cells were cultured in RPMI-1640 medium containing 10% FBS, 100 IU/ml penicillin-streptomycin. Cells were cultured at 37°C in a humidified environment with 5% CO_2_. During the culture of MCF-7/Adr cells, 1 μg/ml of DOX was added to maintain drug resistance. DOX treatment was stopped 2 weeks before the experiment.

### 2.3 MTT assay

Cell viability was detected by 3-(4,5-Dimethylthiazol-2-yl)-2,5-diphenyltetrazolium bromide (MTT) assay. The cells were seeded in 96-well plates at 8 × 10^3^ cells/well, and then exposed to different concentrations of the indicated compounds (10 μl) for 48 h. 20 μl MTT was added to each well for 4 h. Optical density (OD) values per well were determined at 570 nm and 630 nm using a SpectraMax Plus384 Molecular Devices (Molecular Devices, LLC).

### 2.4 RNA-seq

Total RNA was extracted with Trizol from MCF-7, MCF-7/Adr and MCF-7/Adr treated with 8 μM of GA5. RNA purity and concentration were measured using Nanophotometer Pearl (IMPLEN, CA, United States). Preparation of RNA library and transcriptome sequencing was conducted by Novogene Co., LTD. (Beijing, China). The significance analysis of gene expression difference was carried out, and the difference of gene expression under two conditions or multiple conditions was compared using statistical methods, and the specific genes related to the conditions were found out. Genes with adjusted *p*-value < 0.05 and |log2(FoldChange)| > 0 were considered as differentially expressed.

### 2.5 Cell infection

Stable BMP6 over-expression and knockdown cell clones were generated by lentivirus infection. All lentiviruses were obtained from Genechem (Shanghai, China). The infection multiple of infection (MOI) was 20 according to the instructions, and the infection time was 12 h. Before the next experiment, stable-infected cells were screened with 1 μg/ml puromycin for 2 weeks, and the overexpression and knockdown effects were detected by qRT-PCR and western blot analysis.

### 2.6 qRT-PCR

After the cells were collected, total RNA was extracted by TRIzol reagent, and then reverse transcribed into cDNA with a RevertAid First Strand cDNA Synthesis Kit. The target gene was amplified with PowerUp^TM^ SYBR^TM^ Green Master Mix and ABI PRISM^®^ 7500 Real-time PCR system (Applied Biossystems, United States), with β-actin as a control. Primers were purchased from Invitrogen (United States), and the primer sequences are shown in Table 1.

**TABLE 1:** Sequence of qRT-PCR primers used in the research.

Application	Gene	Sequence (5′-3′)
qRT-PCR	BCL2A1	Forward:5′-GGATAAGGCAAAACGGAGGCTG-3′
		Reverse: 5′-CAG​TAT​TGC​TTC​AGG​AGA​GAT​AGC-3′
	ABCB1	Forward: 5′-GCT​GTC​AAG​GAA​GCC​AAT​GCC​T-3′
		Reverse: 5′-TGC​AAT​GGC​GAT​CCT​CTG​CTT​C-3′
	ERCC1	Forward:5′-GCTGGCTAAGATGTGTATCCTGG-3′
		Reverse: 5′-ATC​AGG​AGG​TCC​GCT​GGT​TTC​T-3′
	GSTP1	Forward:5′-TGGACATGGTGAATGACGGCGT-3′
		Reverse: 5′-GGT​CTC​AAA​AGG​CTT​CAG​TTG​CC-3′
	CYP1A2	Forward:5′-TCATCCTGGAGACCTTCCGACA-3′
		Reverse: 5′-GCC​ACT​GGT​TTA​CGA​AGA​CAC​AG-3′
	β-actin	Forward:5′-CACCATTGGCAATGAGCGGTTC-3′
		Reverse: 5′-AGG​TCT​TTG​CGG​ATG​TCC​ACG​T-3′
	TENM4	Forward:5′-GTCACCAACATCCTAGAGCTGAG-3′
		Reverse: 5′-GTT​GCT​GTC​AGA​AAG​GAA​GAC​GG-3′
	PCDHA12	Forward:5′-CAAGCCTTCAGCTGTCTCGAGA-3′
		Reverse: 5′-AGA​ATG​CCA​GCC​TCC​TCT​AGG​T-3′
	PLPPR4	Forward:5′-TCCTTCCTCAGACGAGCTGTCA-3′
		Reverse: 5′-CAC​AGT​CAG​AAA​GTA​AGG​TGC​TTG-3′
	HIST1H1B	Forward:5′-CCGAAAAAGGCAACCAAGAGTCC-3′
		Reverse: 5′-GTT​TTC​ACA​CGC​CAG​CTT​CCT​AC-3′
	HFM1	Forward:5′-CCAGCACTGCTATTCCAATGCG-3′
		Reverse: 5′-CAC​TGG​TCT​ATG​GCT​CTC​ATC​C-3′
	BMP6	Forward:5′-CCGACAACAGAGTCGTAATCGC-3′
		Reverse: 5′-CTG​CCA​TCC​CAG​GTC​TTG​GAA​A-3′
	ANGPTL4	Forward:5′-GATGGCTCAGTGGACTTCAACC-3′
		Reverse: 5′-TGC​TAT​GCA​CCT​TCT​CCA​GAC​C-3′
	TLE3	Forward:5′-CCACCATGAACTCGATCACAGAG-3′
		Reverse: 5′-CTT​GGC​TTC​CAT​GCT​GTA​GTC​C-3′
	NDRG1	Forward:5′-ATCACCCAGCACTTTGCCGTCT-3′
		Reverse: 5′-GAC​TCC​AGG​AAG​CAT​TTC​AGC​C-3′
	GPR78	Forward:5′-GACCTTCCTCATCTGCTTTGCC-3′
		Reverse: 5′-CCT​TGC​TGT​AGG​TCA​GGC​ACT​T-3′
	CAVIN-2	Forward:5′-GCGGTCAAAGAGCGCATGGATA-3′
		Reverse: 5′-AAA​CAC​GCT​GGC​AGG​GAT​CTC​A-3′

### 2.7 Western blot analysis

Cells were collected and placed on ice, and lysed by RIPA lysis buffer (Beyotime, China) containing protease inhibitors and phosphatase inhibitors. Equal amounts of cell lysate (20 μg of protein) were separated by sodium dodecyl sulfate-polyacrylamide gel electrophoresis (SDS-PAGE) and were transferred to polyvinylidene difluoride (PVDF) membrane (Millipore, United States). Western blot analysis was performed with the following primary antibodies: anti-β-tubulin, anti-p44/42 MAPK (Erk1/2) and anti-MDR1/ABCB1 (1:1000 dilution; Cell Signaling Technology, United States), anti-BMP6, anti-TLE3 and anti-NDRG1 (1:5000 dilution; Abcam, United Kingdom), anti-Phospho-ERK1/2 (Thr202/Tyr204) (1:1000 dilution; Genxspan, China).

### 2.8 Xenografted animal studies

All animal experiments in this study were carried out following the guidance of the Animal Care and Use Committee of Kunming Medical University (Kunming, China). All the animal protocol and procedures were approved by the Ethics Review Committee for Animal Experimentation of Kunming Medical University. Four-week-old female nude mice were purchased from Beijing Vital River Laboratory Animal Technology Co., Ltd. and allowed 1 week to adapt the laboratory environment. MCF-7 cells and MCF-7/Adr cells were suspended in 1:1 PBS and Matrigel (354234, Corning) to a concentration of 1×10^7^ cells/ml. The cell suspension (0.2 ml/mice) was inoculated under the right armpit of nude mice. 32 mice respectively inoculated with MCF-7 cells and MCF-7/Adr cells were divided into four groups: control group, DOX group (2.5 mg/kg), GA5 low dose (1 mg/kg) group and GA5 high dose (5 mg/kg) group. When the volume of tumors reached above 100 mm^3^, control and DOX group mice were administered physiological saline and DOX respectively once every 2 days by intraperitoneal injection. GA5 group mice was administered by gavage with 1 mg/kg and 5 mg/kg of GA5 once a day for 6 days a week continuously. Subcutaneous tumors were measured twice a week with an electronic caliper, and the tumor size was calculated according to the formula: 1/2 (length × width^2^). All mice were sacrificed after 3 weeks of intervention. Tumor specimens were dissected for endpoint measurements, weighed, and subsequently fixed in formalin for immunohistochemistry (IHC) analysis.

### 2.9 Immunohistochemistry analysis

After paraffin-embedding, the tissue sections were placed in citrate antigen retrieval buffer (PH6.0) for antigen retrieval. Next, endogenous peroxidase was blocked with 3% H_2_O_2_, and then the tissue was evenly covered with 3% BSA and blocked at room temperature for 30 min. Then primary antibody was added and incubated overnight at 4°C (BMP6, 1:100, Abcam, ab155963; P-gp, 1:1200, Abcam, ab170904; p-ERK, 1:200, Cell Signaling Technology, #5726). After washing the sections with PBS, the corresponding secondary antibodies (HRP-labeled) were added and incubated at room temperature for 50 min. After washing with PBS again, freshly prepared diaminobenzidine (DAB) was used for color development. The positive was brownish yellow, and the color development was terminated by washing with tap water. Finally, the sections were counterstained with hematoxylin, dehydrated with graded alcohol, and mounted for microscopic examination. Three tumor tissues were randomly selected from each group to make a total of six slices, and three high-power fields of each slice were randomly selected for scanning using caseviewer scanning software, and the positive area rate was analyzed by ImageJ software. Except antibodies, all the other reagents were purchased from Wuhan servicebio technology CO., LTD. (China).

### 2.10 Statistical analysis

Data were represented as the mean ± standard deviation (SD) from three independent experiments. The two-group comparison was analyzed by Student’s *t*-test. Results of multiple groups were compared by one-way ANOVA. The results were assessed for differences by using SPSS 24.0 and GraphPad Prism 8.0 software. Differences were considered statistically significant when *p* < 0.05.

## 3 Results

### 3.1 GA5 exhibited more potent anti-proliferation effect against multidrug resistant breast cancer cells

To identify the MDR property of MCF-7/Adr cells used in our study, the cytotoxicity of five chemotherapeutic drugs was detected in human breast cancer parental cells MCF-7 and the corresponding drug-resistant cells MCF-7/Adr. As shown in Table 2, the chemotherapeutic drugs including DOX, HCPT, NVT, EPI and VP-16 showed cytotoxic effects against MCF-7 cells with IC_50_ values of 1.25, 9.81, 4.36, 0.17 and 92.85 μM, respectively. In drug-resistant MCF-7/Adr cells, the IC_50_ of DOX, HCPT, NVT, EPI and VP-16 was 350.92, 125.28, 349.8, 4.43 and 465.10 μM, respectively. The drug-resistance index of MCF-7/Adr cells to DOX, HCPT, NVT, EPI and VP-16 was 280.74, 12.80, 80.30, 26.06 and 5.00, respectively. The above data proved that MCF-7/Adr cells had multidrug resistant properties. Notably, we further found that GA5 exhibited stronger inhibitory effect against drug-resistant MCF-7/Adr cells with the IC_50_ of 53.83 μM and 14.09 μM in MCF-7 and MCF-7/Adr cells, respectively (Table 3).

**TABLE 2:** The IC_50_ values (48 h) of different chemotherapeutic drugs in MCF-7 and MCF-7/Adr cells and the resistance index of MCF-7/Adr cells to each chemotherapeutic drug.

Drugs	IC50 ± SD (μM),48 h		Resistant index
	MCF-7	MCF-7/Adr	
DOX	1.25 ± 0.05	350.92 ± 11.10^***^	280.74
HCPT	9.81 ± 0.11	125.28 ± 0.09^***^	12.80
NVT	4.36 ± 1.11	349.80 ± 65.60^**^	80.30
EPI	0.17 ± 0.02	4.43 ± 1.01^***^	26.06
VP-16	92.85 ± 3.90	465.10 ± 8.20^***^	5.00

**p<0.01, ***p<0.001, difference versus MCF-7 cells.

**TABLE 3:** Effect of compound GA5 on proliferation of MCF-7 and MCF-7/Adr cells (48 h).

Compound	IC_50_ ± SD (μM)	
	MCF-7	MCF-7/Adr
GA5	53.83 ± 1.51	14.09 ± 2.32***

***p<0.001, difference versus MCF-7 cells.

### 3.2 Screening of genes involved in multidrug resistance of MCF-7/Adr cells and GA5 overcoming drug resistance by RNA-Seq

**FIGURE 2:**
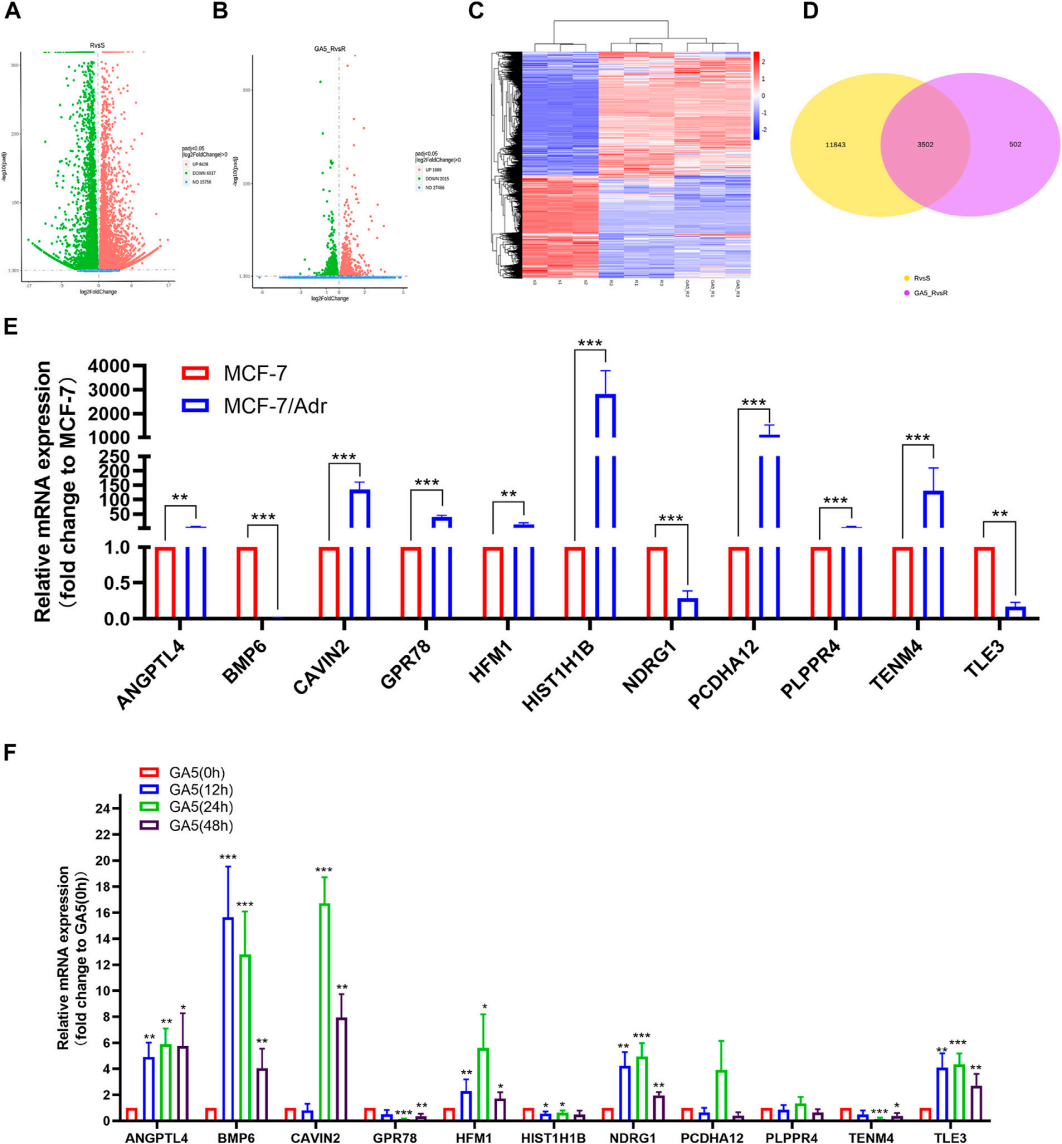
Screening of genes involved in MDR of MCF-7/Adr cells and GA5 overcoming drug resistance by RNA-Seq. **(A)** The volcano plot showing the transcriptome differences between MCF-7 and MCF-7/Adr cells. **(B)** The volcano plot showing the transcriptome differences between GA5 treatment and non-GA5 treatment MCF-7/Adr cells. The abscissa represents the fold change (log2FoldChange) of gene expression in the treatment and control groups. The ordinate represents the significant level (-log10padj or -log10*p*-value) of the gene expression difference between the treatment and control groups, and up-regulated genes were represented by red dots, down-regulated genes were indicated by green dots. **(C)** Differential gene clustering heat map. From left to right were three MCF-7 samples (s1, s2, s3), three MCF-7/Adr samples (R1, R2, R3) and three GA5-treatment MCF-7/Adr samples (GA5_R1, GA5_R2, GA5_R3). **(D)** Venn diagram of differential genes. Yellow represents differential genes between MCF-7 and MCF-7/Adr cells. Purple represents differential genes between GA5 treatment and non-GA5 treatment MCF-7/Adr cells, and pink represents common differential genes. **(E)** mRNA levels of ANGPTL4, BMP6, CAVIN-2, GPR78, HFM1, HIST1H1B, NDRG1, PCDHA12, PLPPR4, TENM4 and TLE3 in MCF-7 and MCF-7/Adr cells. **p < 0.01, ***p < 0.001, difference versus MCF-7 cells. **(F)** mRNA levels of ANGPTL4, BMP6, CAVIN-2, GPR78, HFM1, HIST1H1B, NDRG1, PCDHA12, PLPPR4, TENM4 and TLE3 in MCF-7/Adr cells which were treated with 8 μM of GA5 for indicated time periods. *p < 0.05, **p < 0.01, ***p < 0.001, difference versus GA5 non-treatment group.

To discover differentially expressed genes between the multidrug resistant cells and the parental cells, and to identify the genes related to overcoming MDR by GA5, RNA-Seq was performed in parental cell line MCF-7 and multidrug resistant cell line MCF-7/Adr, and in GA5 treatment and non-GA5 treatment MCF-7/Adr cells. According to Table 4 and the volcano plot in Figures 2A,B, the overall distribution of the differential genes could be preliminarily inferred. There were 8428 up-regulated genes and 6917 down-regulated genes in MCF-7/Adr cells when compared with MCF-7 cells (Figure 2A). Meanwhile, 1989 genes were up-regulated and 2015 genes were down-regulated after GA5 treatment in MCF-7/Adr cells (Figure 2B). Differential gene clustering heat map (Figure 2C) showed the gene expressions and differences between MCF-7 cells and MCF-7/Adr cells, and between GA5 treatment and non-GA5 treatment MCF-7/Adr cells. In this heat map, red indicated high gene expression and blue indicated low gene expression (Figure 2C). The overlap in the middle of the figure (Figure 2D) showed that 3502 genes might be involved in overcoming drug resistance by GA5. Based on the data analysis of RNA-Seq, we queried the information and functions of these genes through Genecard, reviewed several literatures, and finally selected the genes showed in Table 5 for further verification.

**TABLE 4:** Differential gene expression.

Compare	Up	Downn	Threshold
R vs. S	8428	6917	padj<0.05 |log_2_FoldChange|>0.0
GA5_R vs. R	1989	2015	padj<0.05 |log_2_FoldChange|>0.0

R vs. S means MCF-7/Adr cells compared with MCF-7 cells.

GA5_R vs. R means GA5 treatment compared with non-GA5 treatment MCF-7/Adr cells.

**TABLE 5:** Differential genes identified from RNA-Seq analysis.

Gene name	MCF-7/Adr	MCF-7	|Log2 ^FC^|	GA5+MCF-7/Adr	MCF-7/Adr	|Log2 ^FC^|
ANGPTL4	405.25	2347.53	−2.53	433.45	1399.59	1.69
BMP6	56.98	3263.15	−5.83	154.62	60.95	1.34
CAVIN2	1804.80	21.45	6.38	829.85	1930.31	−1.22
GPR78	154.32	24.45	2.66	76.40	165.06	−1.11
HFM1	26.43	0.00	7.08	11.44	28.26	−1.30
HIST1H1B	41.02	0.00	7.71	19.33	43.86	−1.18
NDRG1	9089.69	19678.54	−1.11	26284.68	9721.53	1.43
PCDHA12	79.56	0.00	8.66	36.97	85.08	−1.20
PLPPR4	31.53	0.00	7.33	33.72	12.69	−1.41
TENM4	262.72	0.00	10.39	138.41	281.03	−1.02
TLE3	179.29	2997.71	−4.06	427.87	191.76	1.16

Based on the RNA-Seq analysis, qRT-PCR was further used to determine the mRNA levels of the genes showed in Table 5. The results showed that the mRNA expressions of BMP6, TLE3 and NDRG1 were significantly down-regulated in the MCF-7/Adr cells when compared with the parental MCF-7 cells (Figure 2E). After GA5 treatment in MCF-7/Adr cells, the expressions of the three genes were significantly up-regulated (Figure 2F). Therefore, the three genes including BMP6, TLE3 and NDRG1 were selected for further verification by western blot analysis.

### 3.3 BMP6 protein expression was down-regulated, and GA5 up-regulated BMP6 expression in MCF-7/Adr cells

**FIGURE 3:**
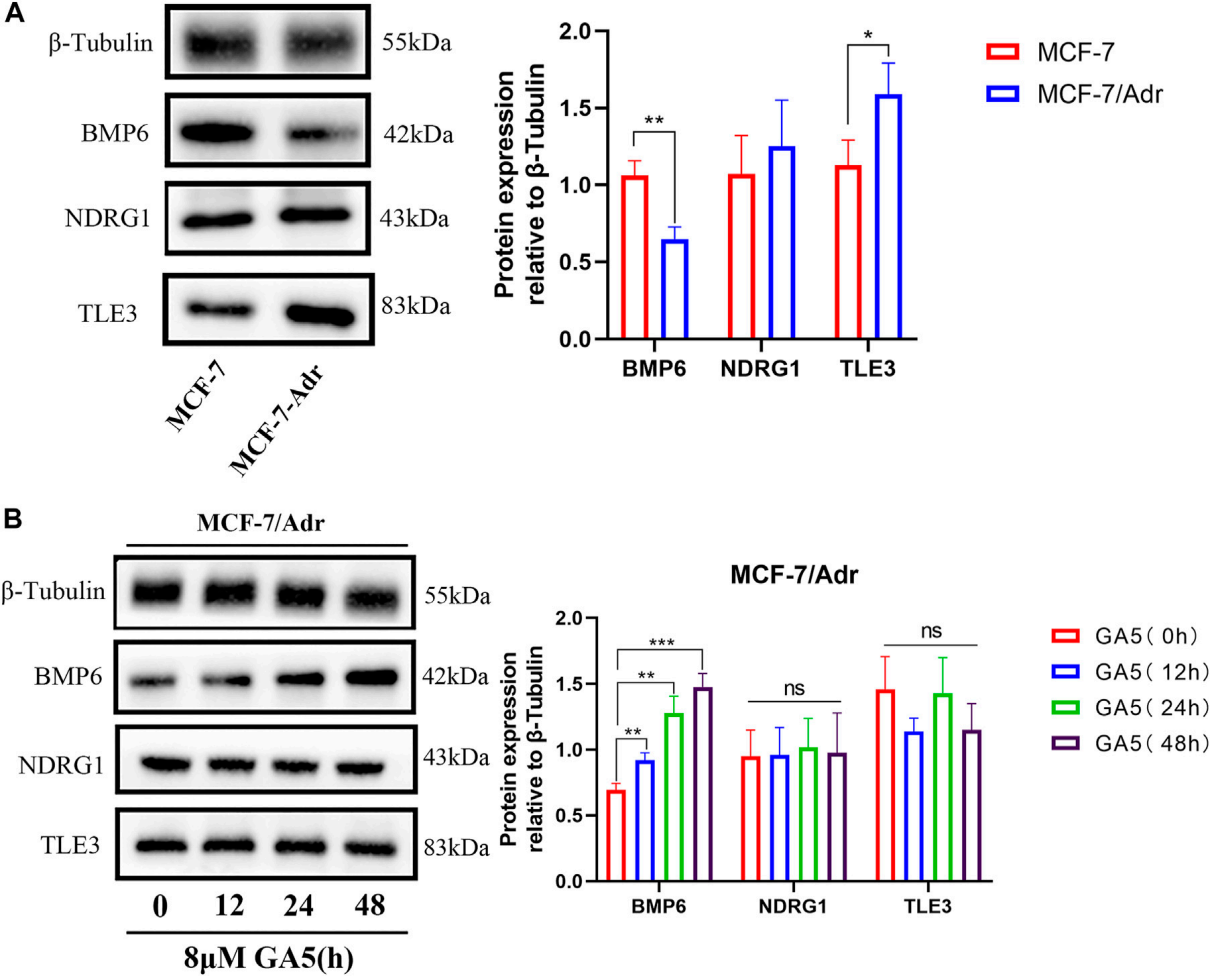
BMP6 protein expression was down-regulated, and GA5 could up-regulate BMP6 expression in the drug-resistant MCF-7/Adr cells. **(A)** Western blot analysis on the expressions of BMP6, TLE3 and NDRG1 proteins in MCF-7 and MCF-7/Adr cells. Blots for indicated protein expressions were semi-quantified using ImageJ software. The data were represented as the means ± SD (*n* = 3). *p < 0.05, **p < 0.01 difference versus MCF-7 cells. **(B)** After MCF-7/Adr cells were treated with 8 μM of GA5 for 0, 12, 24 and 48h, the expression levels of BMP6, TLE3 and NDRG1 proteins were detected by western blot analysis. Blots for indicated protein expressions were semi-quantified using ImageJ software. The data were represented as means ± SD (*n* = 3). Ns: not significant. **p < 0.01, ***p < 0.001 difference versus GA5 non-treatment group.

The protein expressions of BMP6, TLE3 and NDRG1 in MCF-7 and MCF-7/Adr cells, and the effect of GA5 on these protein expressions in MCF-7/Adr cells were determined by western blot analysis. Consistent with the qRT-PCR results, BMP6 protein expression was down-regulated in the drug-resistant MCF-7/Adr cells when compared with the parental cells (Figure 3A). However, TLE3 protein expression was higher in the drug-resistant MCF-7/Adr cells when compared with the parental cells, and NDRG1 protein expression in MCF-7 and MCF-7/Adr cells was not significantly different, which was inconsistent with the qRT-PCR results (Figure 3A). Moreover, after GA5 treatment for 12, 24 and 48 h in MCF-7/Adr cells, the expression of BMP6 protein was up-regulated, which was consistent with the results of qRT-PCR (Figure 3B). However, GA5 treatment did not change the protein expressions of NDRG1 and TLE3 (Figure 3B). Based on the above results, we hypothesized that BMP6 down-regulation might be involved in the development of MDR in MCF-7/Adr cells and GA5 overcame MDR of MCF-7/Adr cells might be attributed to BMP6 up-regulation.

### 3.4 Overexpression of BMP6 sensitized MCF-7/Adr cells to chemotherapeutic drugs

**FIGURE 4:**
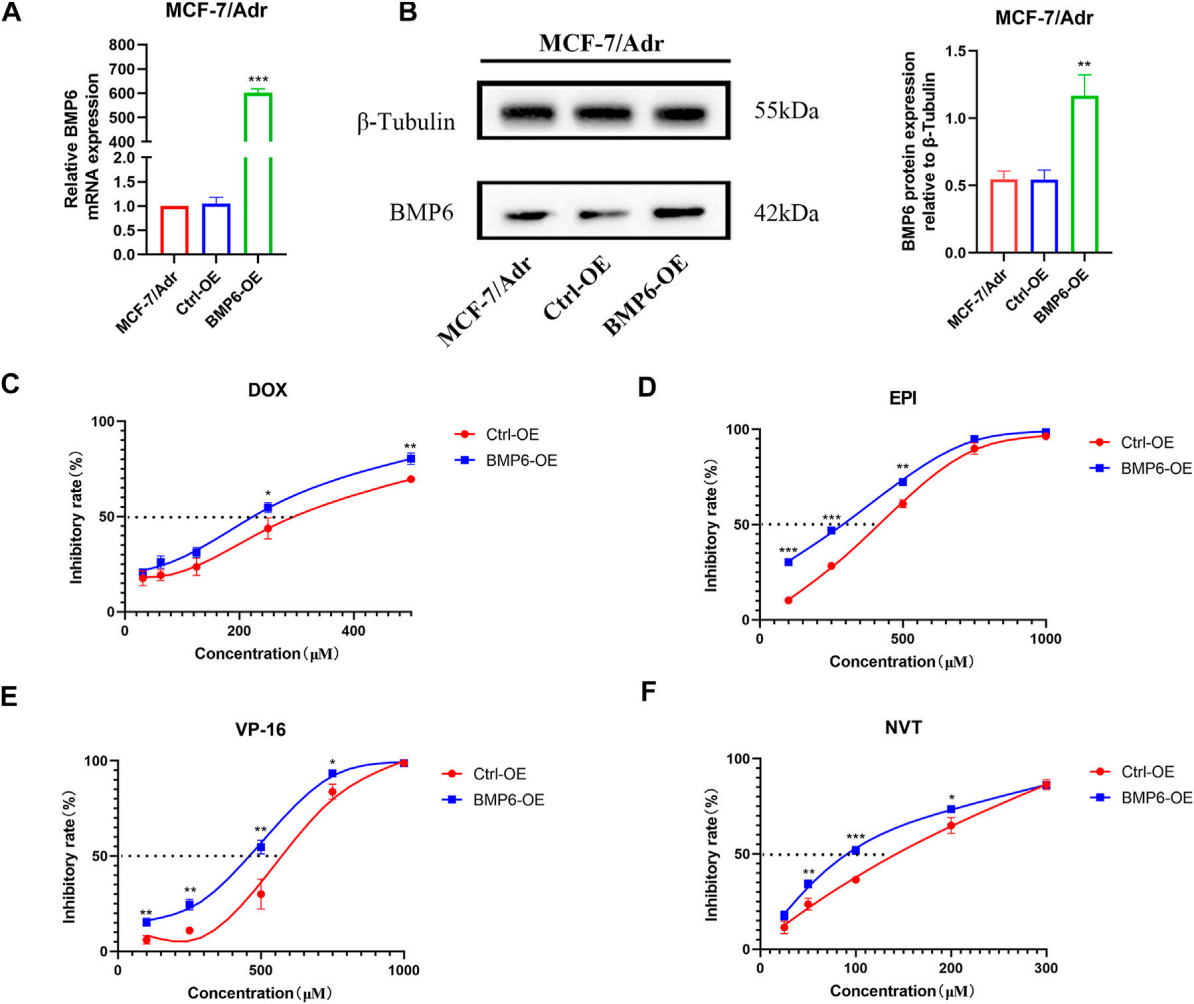
Overexpression of BMP6 sensitized MCF-7/Adr cells to chemotherapeutic drugs. **(A)** qRT-PCR was used to detect the expression of BMP6 mRNA expression in MCF-7/Adr cells after lentivirus infection. **(B)** Western blot was used to detect BMP6 protein expression in MCF-7/Adr cells after lentivirus infection. Data were presented as the means ± SD (*n* = 3). **p < 0.01, ***p < 0.001 difference versus Ctrl-OE MCF-7/Adr cells. **(C–F)** Overexpression of BMP6 sensitized MCF-7/Adr cells to DOX, EPI, VP-16 and NVT. The experiment was repeated at least three times. Data shown were means ± SD. Proliferation inhibition rate (%) = (OD value of control group- OD value of treated group)/OD value of control group × 100%. **p < 0.01, ***p < 0.001 difference versus Ctrl-OE MCF-7/Adr cells.

To investigate whether the down-regulation of BMP6 was involved in the development of MDR, BMP6 was overexpressed by lentivirus infection in MCF-7/Adr cells. The infection efficiency was verified by qRT-PCR and western-blot analysis (Figures 4A,B). Meanwhile, cell sensitivity to chemotherapeutic drugs was determined in MCF-7/Adr cells and BMP6 overexpressed MCF-7/Adr cells. Compared with the empty vector group (Ctrl-OE), overexpression of BMP6 improved cell sensitivity to DOX, EPI, VP-16 and NVT, suggesting that the down-regulation of BMP6 was associated with the development of MDR in MCF-7/Adr cells.

### 3.5 GA5 overcame multidrug resistance in MCF-7/Adr cells by up-regulating BMP6 protein expression

**FIGURE 5:**
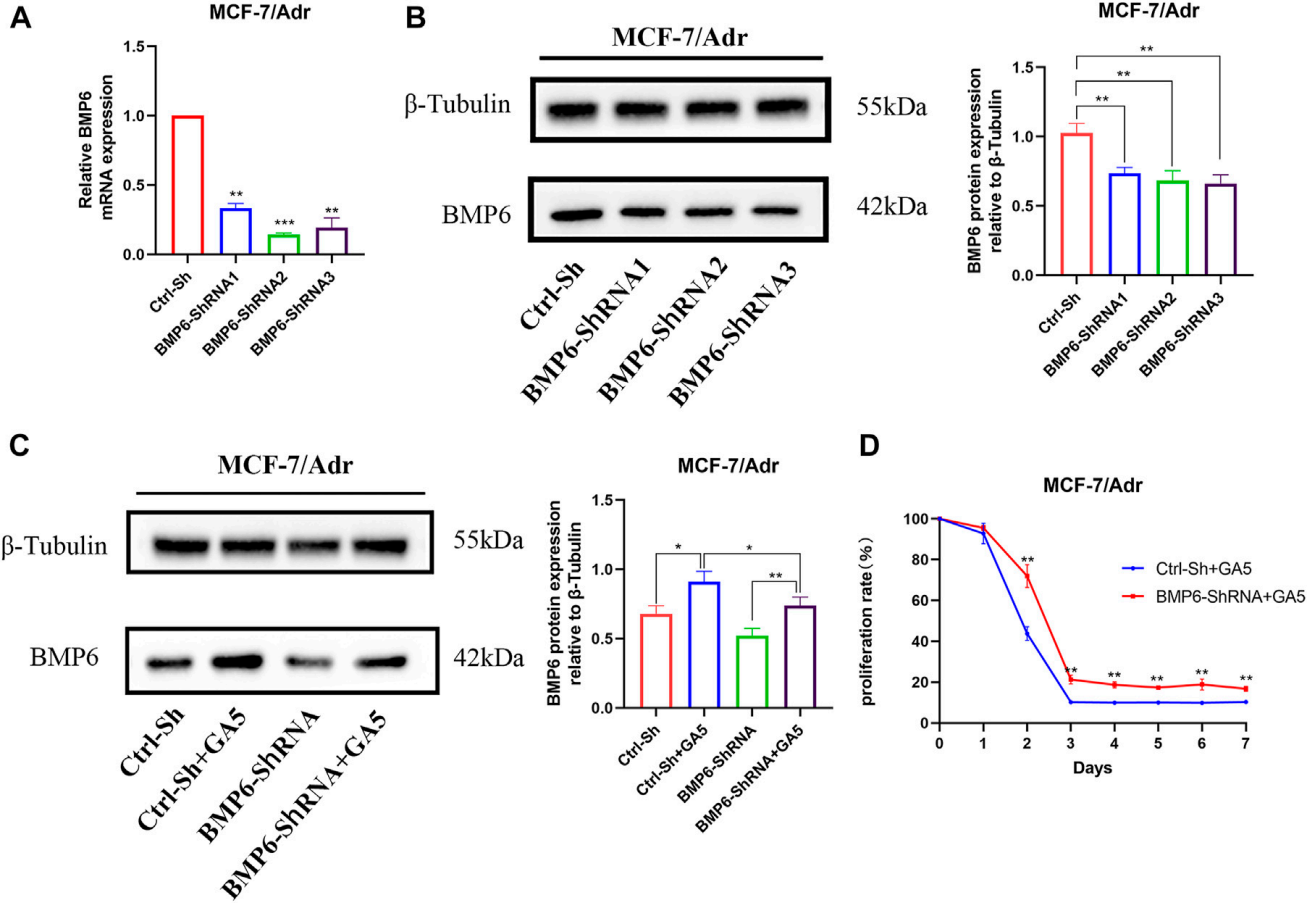
GA5 overcame MDR of MCF-7/Adr cells by up-regulating the expression of BMP6 protein. **(A)** qRT-PCR was used to detect the mRNA level of BMP6 in MCF-7/Adr cells after BMP6-ShRNA lentivirus infection. **(B)** Western blot was used to detect BMP6 protein expression in MCF-7/Adr cells after BMP6-ShRNA lentivirus infection. Data were represented as the means ± SD (*n* = 3). **p < 0.01, ***p < 0.001 difference versus Ctrl-Sh MCF-7/Adr cells. **(C)** BMP6 knockdown effectively inhibited GA5-induced BMP6 expression. Blots for indicated protein expressions were semi-quantified using ImageJ software. Data shown were means ± SD (*n* = 3). *p < 0.05, **p < 0.01 difference versus Ctrl-Sh MCF-7/Adr cells. **(D)** BMP6 knockdown partially reversed the anti-proliferative effect of GA5 in MCF-7/Adr cells. The experiments were repeated at least three times, and the data shown were means ± SD. **p < 0.01 difference versus GA5-treated Ctrl-Sh MCF-7/Adr cells.

To explore whether GA5 overcame MDR in MCF-7/Adr cells by upregulating BMP6, BMP6 was knocked down by lentivirus infection in MCF-7/Adr cells. The infection efficiency was verified by qRT-PCR and western-blot (Figures 5A,B). Compared with the empty vector group (Ctrl-Sh), the expression of BMP6 in the BMP6-shRNA infection groups was reduced (Figures 5A,B). Meanwhile, BMP6 knockdown effectively decreased GA5-induced BMP6 expression (Figure 5C), and partially reversed the anti-proliferative effect of GA5 in MCF-7/Adr cells (Figure 5D), indicating that GA5 overcame MDR of MCF-7/Adr cells was attributed to BMP6 up-regulation.

### 3.6 GA5 inhibited the activation of ERK signaling pathway by upregulating BMP6

**FIGURE 6:**
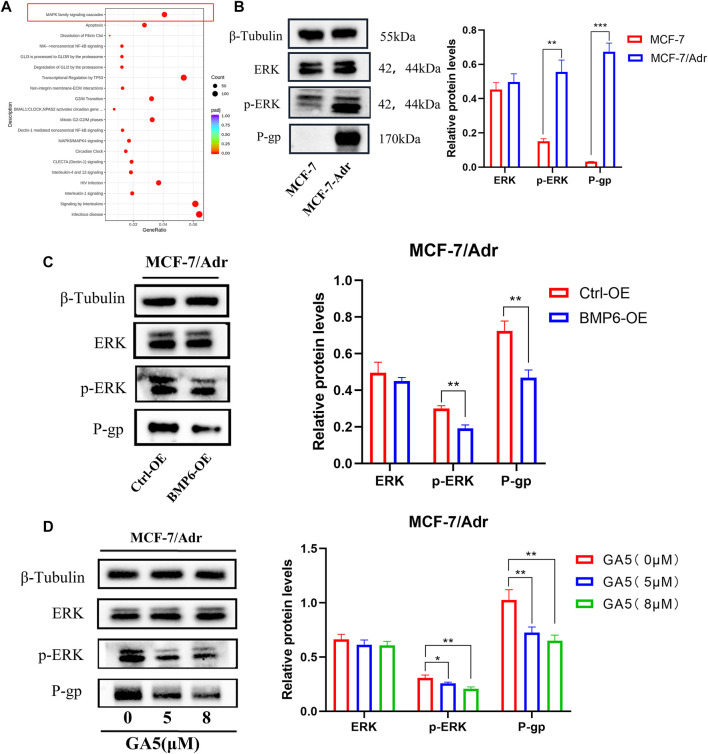
BMP6 overexpression or GA5 treatment in MCF-7/Adr cells decreased the phosphorylation of ERK and the expression of P-gp. **(A)** The reaction and biological pathway changes after GA5 treatment were analyzed by Reactome functional enrichment in MCF-7/Adr cells. **(B)** The phosphorylation of ERK and P-gp expression in MCF-7 and MCF-7/Adr cells was detected by western blot. **p < 0.01, ***p < 0.001 difference versus MCF-7 cells. **(C)** The phosphorylation of ERK and P-gp expression were detected in Ctrl-OE and BMP6-OE MCF-7/Adr cells by western blot. **p < 0.01, difference versus Ctrl-OE MCF-7/Adr cells. **(D)** The phosphorylation of ERK and P-gp expression after different concentrations of GA5 (5 μM and 8 μM) treatment in MCF-7/Adr cells were detected by western blot. Blots for indicated protein expressions were semi-quantified using ImageJ software, the densities of ERK and P-gp were calculated and normalized with β-Tubulin, and the densities of p-ERK were calculated and normalized with ERK. Data shown were means ± SD (*n* = 3). *p < 0.05, **p < 0.01, difference versus Ctrl-OE MCF-7/Adr cells.

The functional enrichment results of Reactome (https://reactome.org/) showed that the genes of MAPK family cascade signaling were down-regulated after GA5 intervention in MCF-7/Adr cells (Figure 6A). The study of Lian et al. reported that the low expression of BMP6 in parental MCF-7 cells was related to the activation of ERK signaling pathway and the high expression of P-gp/MDR1 protein [16]. Therefore, we speculated that the inhibition of ERK signaling pathway and the expression of P-gp protein by upregulating BMP6 might be one of the mechanisms by which GA5 overcame MDR in MCF-7/Adr cells. Our results showed that the phosphorylation of ERK and the expression of P-gp protein were significantly increased in MCF-7/Adr cells when compared with MCF-7 cells (Figure 6B). Overexpression of BMP6 in MCF-7/Adr cells inhibited the phosphorylation level of ERK and decreased the expression of P-gp protein (Figure 6C). Meanwhile, different concentrations of GA5 treatment in MCF-7/Adr cells inhibited the phosphorylation level of ERK and down-regulated the expression of P-gp protein as well (Figure 6D). These results suggested that GA5 could inhibit the activation of ERK signaling pathway and reduce the expression of P-gp protein by increasing BMP6 expression, and thereby overcome MDR in MCF-7/Adr cells.

### 3.7 The antitumor activity of GA5 in nude mice bearing MCF-7 and MCF-7/Adr xenografts

**FIGURE 7:**
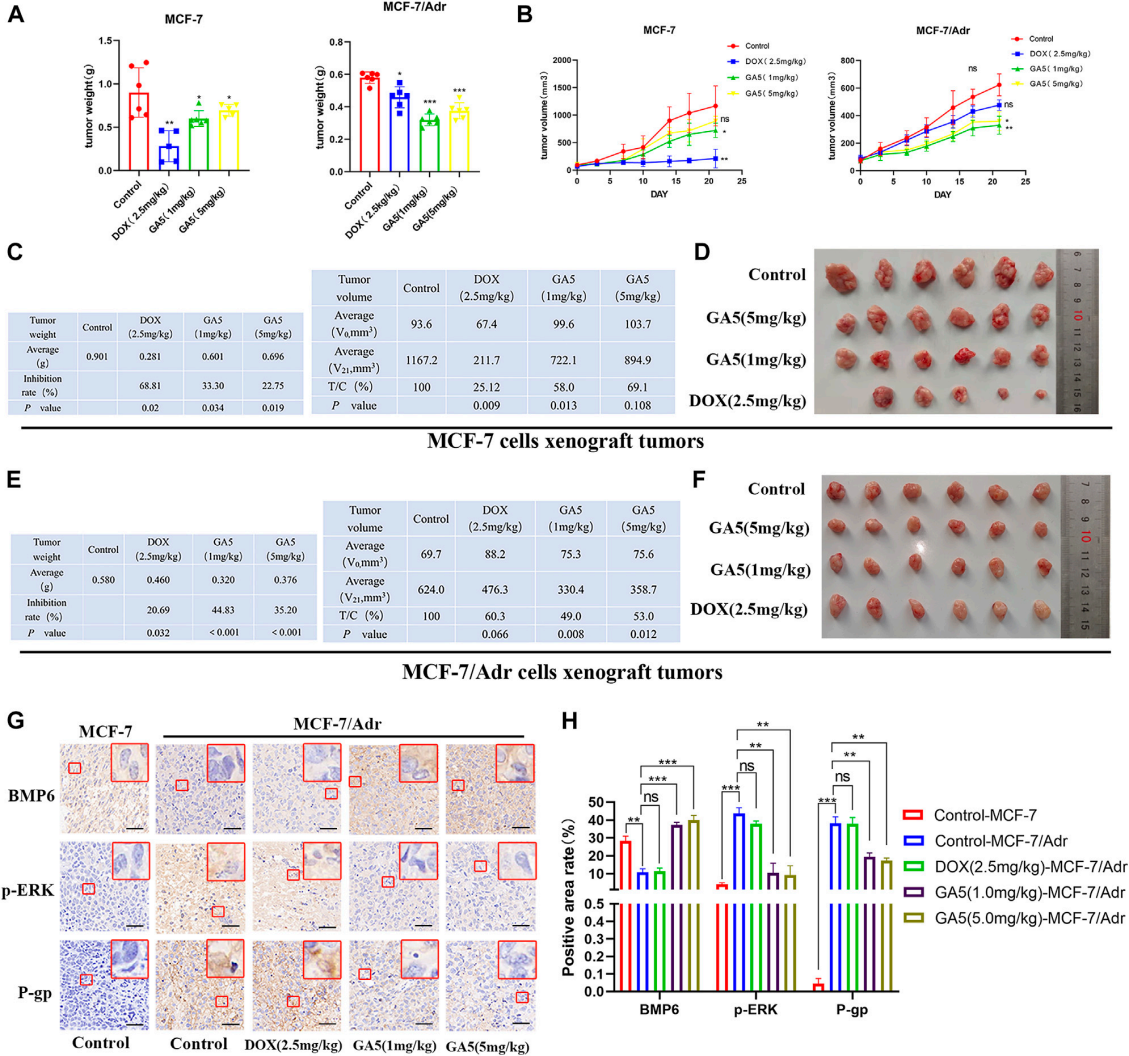
Study on the *in vivo* antitumor activity of GA5 in MCF-7 and MCF-7/Adr xenografts. **(A)** The effects of DOX and GA5 on tumor weight (mg) in MCF-7 and MCF-7/Adr xenografts in nude mice (*n* = 6, among which the DOX group in MCF-7 xenografted nude mice *n* = 5, one died on the 14th day). Data were represented as the means ± SD. *p < 0.05, **p < 0.01, ***p < 0.001 difference versus control group. **(B)** The effects of DOX and GA5 on tumor growth in MCF-7 and MCF-7/Adr xenografts. Subcutaneous tumors were measured twice a week with an electronic caliper, and the tumor volume was calculated. Data were represented as the means ± SD. Ns: not significant. *p < 0.05, **p < 0.01 difference versus control group. **(C)** Tumor inhibition rate and relative tumor proliferation rate were calculated according to the data of tumor weight and volume in MCF-7 xenografts. **(D)** Pictures of the excised MCF-7 tumors at the end of the experiment. (*n* = 6, among which the DOX group in MCF-7 xenografted nude mice *n* = 5, one died on the 14th day) **(E)** Tumor inhibition rate and relative tumor proliferation rate were calculated according to the data of tumor weight and volume in MCF-7/Adr xenografts. **(F)** Pictures of the excised MCF-7/Adr tumors at the end of the experiment. **(G)** Immunohistochemical staining was used to analyze the expression of BMP6, p-ERK and P-gp proteins in MCF-7 and MCF-7/Adr nude mice xenografts. Scale bar = 20 µm. **(H)** Three high-power fields were randomly selected for each slice to scan with Caseviewer scanning software, and the positive area rate was analyzed by ImageJ software. Data shown were means ± SD (*n* = 18). Ns: not significant. **p < 0.01, ***p < 0.001 difference versus control group.

In order to investigate whether GA5 could reverse MDR *in vivo*, the antitumor efficacy of GA5 was determined in nude mice bearing MCF-7 and MCF-7/Adr xenografts. The results showed that the chemotherapeutic drug DOX (2.5 mg/kg) significantly inhibited the growth of MCF-7 transplanted tumors in nude mice (Figures 7A–D). The tumor inhibition rate and relative tumor proliferation rate (T/C%) of DOX (2.5 mg/kg) was 68.81% and 25. 12%, respectively (Figure 7C). However, the tumor inhibition rate and T/C% of the same dose of DOX (2.5 mg/kg) on the growth of MCF-7/Adr xenografted tumors was 20.69% and 60.30%, respectively (Figure 7E), indicating that the growth of MCF-7/Adr xenografted tumors was not effectively inhibited by DOX (2.5 mg/kg). The above data validated that the *in vivo* model is a DOX-resistant xenograft model. Moreover, in MCF-7 xenografts, the inhibition rate of GA5 at 1 mg/kg and 5 mg/kg on the growth of tumors was 33.30% and 22.75%, respectively, and T/C% was 58.00% and 69.10%, respectively (Figures 7A–D). Notably, the inhibition rate of GA5 at 1 mg/kg and 5 mg/kg on the growth of MCF-7/Adr xenografted tumors was 44.83% and 35.20%, respectively, and the T/C% were 49.00% and 53.00%, respectively (Figures 7E,F). These data demonstrated that the inhibitory effect of GA5 on DOX-resistant MCF-7/Adr xenografted tumors was stronger than that on the sensitive MCF-7 xenografted tumors, which was consistent with the results of *in vitro* study.

Meanwhile, the expression levels of BMP6, p-ERK and P-gp proteins in tumor tissues were analyzed by immunohistochemical staining. The results showed that compared with MCF-7 xenografted tumors, the expression of BMP6 was down-regulated, while the expression levels of p-ERK and P-gp were up-regulated in MCF- 7/Adr tumor tissues (Figures 7G,H). In MCF-7/Adr xenografts, DOX (2.5 mg/kg) treatment had no significant effect on the expression of these proteins (Figures 7G,H). In contrast, GA5 (1 mg/kg and 5 mg/kg) treatment significantly increased BMP6 expression, and decreased p-ERK and P-gp expressions in MCF-7/Adr xenografts. These data further indicated that GA5 overcame MDR in MCF-7/Adr xenografts might be through the inhibition of ERK signaling pathway and P-gp expression by upregulating BMP6 protein level.

## 4 Discussion

Chemoresistance represents a major obstacle in breast cancer treatment (Abd El-Aziz et al., 2021; Cao et al., 2021). To prolong survival time of breast cancer patients, it is critical to identify new strategy to overcome chemoresistance (Tang et al., 2016; Jabbarzadeh Kaboli et al., 2020). GA5 is a new tetracyclic diterpenoid selected from a series of synthesized gibberellin derivatives. Our previous study showed that GA5 had potent anti-tumor activity *in vitro* and *in vivo* with low toxicity (Zhang et al., 2012; Shen and Tang 2017). The median lethal dose (LD_50_) of GA5 in mice by intragastric administration was 4.19 g/kg, while the effective anti-tumor dose was lower than 20 mg/kg (Zhang et al., 2012). Moreover, we found that GA5 exhibited stronger inhibitory effect against MCF-7/Adr breast cancer cells, indicating that GA5 could overcome DOX-resistance of breast cancer cells (Mo et al., 2016).

MCF-7/Adr cells are DOX-induced MDR breast cancer cell lines, which have been widely used as a MDR model in the study of tumor chemotherapy resistance (Ke et al., 2011; Li et al., 2013). In the present study, we demonstrated that MCF-7/Adr cells exhibited different degrees of resistance to the chemotherapeutic drugs including DOX, HCPT, NVT and EPI, indicating that MCF-7/Adr cells used in our study had MDR characteristics. Notably, we found that GA5 had stronger inhibitory effect on MCF-7/Adr cells, further demonstrating that GA5 could overcome MDR of MCF-7/Adr cells. Consistently, in MCF-7 and MCF-7/Adr xenografts, GA5 inhibited MCF-7/Adr tumor growth more significantly when compared with MCF-7 xenografted tumors. These data proved that GA5 could reverse MDR in MCF-7/Adr breast cancer both *in vitro* and *in vivo*. In the present study, the underlying mechanisms involved in the MDR of MCF-7/Adr cells and by which GA5 overcame MDR in MCF-7/Adr cells were explored.

RNA-Seq is a powerful method to study the expression changes of the whole transcriptome of genes in cells or tissues after drug action, which can comprehensively understand the drug effect on the whole transcriptome of genes and accelerate the process of discovering drug targets (Wang et al., 2009; Khatoon et al., 2014). Here, RNA-Seq analysis between the drug-resistant cell line MCF-7/Adr and the corresponding parental cell line MCF-7 was performed to screen for genes closely related to drug resistance. Based on the data of RNA-Seq, qRT-PCR and western blot were used to validate the mRNA and protein levels of selected genes. The results showed that BMP6 expression was down-regulated in MCF-7/Adr cells, and GA5 significantly increased BMP6 expression in MCF-7/Adr cells. Based on these results, we speculated that the down-regulation of BMP6 expression was related to the development of MDR in MCF-7/Adr cells, and GA5 overcome MDR in MCF-7/Adr cells might be through up-regulating BMP6 expression.

Previous studies reported that BMP6 played an important role in the occurrence and development of various tumors such as breast cancer, prostate cancer, pleurioma and non-small cell lung cancer (NSCLC) (Clement et al., 1999; Alarmo and Kallioniemi 2010; Lian et al., 2013). As a tumor suppressor gene, low expression of BMP6 led to breast cancer progression. In addition, the down-regulation of BMP6 enhanced the drug resistance of breast cancer cells (Lian et al., 2013; Liu et al., 2014). In NSCLC, BMP6 mRNA and protein expressions in tumor tissues was significantly reduced when compared with the adjacent normal lung tissues. Analysis with the Kaplan-Meier plotter database revealed that patients with NSCLC with low BMP6 mRNA expression had a reduced overall survival rate. The active BMP6 protein significantly inhibited cell proliferation in H460, H1299, A549 and H520 cells (Xiong et al., 2019). Moreover, studies showed an association between BMP6 and skeletal metastases in prostate cancer (Bentley et al., 1992; Autzen et al., 1998). BMP6 expression was detected in the prostate tissue of over 50% of patients with clinically defined metastatic prostate adenocarcinoma, but was not detected in non-metastatic or benign prostate samples or in ocular melanoma tissue (Autzen et al., 1998). However, little is known about how BMP6 expression is regulated and its mechanisms in breast cancer drug resistance. Liu *et al.* found that DNA methylation level of BMP6 in the drug-resistant cell line MCF-7/ADR was significantly increased when compared to their parental cells MCF-7, suggesting that reduced BMP6 expression by DNA methylation contributes to drug resistance in breast cancer cells (Liu et al., 2014).

Our present study aimed to evaluate the relationship between BMP6 and MDR in MCF-7/Adr cells. We established BMP6 knockdown and overexpression cellular models in MCF-7/Adr cells by lentivirus infection. The results showed that BMP6-overexpressed MCF-7/Adr cells were more sensitive to chemotherapeutic drugs including DOX, VP-16, EPI and NVT, indicating that up-regulation of BMP6 could reduce drug resistance and improve the sensitivity to chemotherapeutic drugs in drug-resistant cells. In order to verify whether GA5 overcame MDR by up-regulating the expression of BMP6, BMP6 was knocked down in MCF-7/Adr cells and the effects of GA5 on the cell proliferation was detected. The results showed that BMP6 knockdown decreased GA5-induced BMP6 upregulation in MCF-7/Adr cells, and partially reversed the inhibitory effect of GA5 on cell proliferation, indicating that GA5 overcame MDR of MCF-7/Adr cells by up-regulating BMP6 expression. However, we believed that upregulating BMP6 was not the only mechanism involved in GA5 overcame the MDR of MCF-7/Adr cells. There might be other mechanisms which are needed to be further explored.

In order to study the specific mechanism by which GA5 overcame the MDR of MCF-7/Adr cells by regulating BMP6 expression, pathway enrichment analysis was further performed. Our data showed that BMP6 was correlated well with ERK signaling pathway. Consistently with the literature, it was reported that the low expression of BMP6 in cells was related to the activation of ERK signaling pathway and the high expression of P-gp/MDR1 protein (Lian et al., 2013). In drug-resistant cells of tumors, the ERK signaling pathway is activated, which can increase the expression of P-gp protein, thereby increasing the drug resistance of tumor cells (Guo et al., 2016; Liu et al., 2017; Chai et al., 2020). P-gp protein is the most characterized protein in the ABC transporter family, encoded by MDR gene 1 (MDR1 or ABCB1) (Ambudkar et al., 1999; Mirzaei et al., 2022). Study has shown that increased efflux of multiple chemotherapeutic drugs, such as DOX, daunorubicin, vincristine, vinblastine, methotrexate and mitoxantrone by P-gp was implicated in the development of resistance of cancer cells (Syed et al., 2017). Several studies have confirmed that the high expression of P-gp was the main factor for the poor prognosis of tumor chemotherapy (Alfarouk et al., 2015; Sachs et al., 2019).

To this end, we detected the phosphorylation level of cellular ERK protein and the expression of drug resistance-related transporter P-gp in MCF-7 and MCF-7/Adr cells. The results showed that the phosphorylation of ERK and the expression of P-gp were increased in the resistant MCF-7/Adr cells when compared with the sensitive MCF-7 cells. Furthermore, overexpression of BMP6 significantly decreased the phosphorylation level of ERK and the expression of P-gp in MCF-7/Adr cells. Similar with the result of overexpression of BMP6, GA5 treatment significantly decreased the phosphorylation level of ERK and the expression of P-gp in MCF-7/Adr cells. In MCF-7 and MCF-7/Adr xenografts, the expression levels of BMP6, p-ERK and P-gp proteins were analyzed by immunohistochemical staining of tumor tissues, and the results were consistent with the results from *in vitro* study.

In conclusion, our present study demonstrated that the MDR of MCF-7/Adr cells was closely related to the low expression of BMP6. GA5, a gibberellin derivative, could inhibit the activation of ERK signaling pathway and reduce the expression of P-gp protein by increasing BMP6 expression, and thereby overcome the MDR in MCF-7/Adr cells. The results of our study revealed the molecular mechanisms by which GA5 overcame MDR in breast cancer cells, and provided evidence in supporting the development of GA5 to be a promising agent for overcoming MDR in clinical cancer therapy in the future.

## Data Availability

All relevant data is contained within the article: The original contributions presented in the study are included in the article/supplementary material, further inquiries can be directed to the corresponding authors.
